# Vitamin E in Sarcopenia: Current Evidences on Its Role in Prevention and Treatment

**DOI:** 10.1155/2014/914853

**Published:** 2014-07-06

**Authors:** Shy Cian Khor, Norwahidah Abdul Karim, Wan Zurinah Wan Ngah, Yasmin Anum Mohd Yusof, Suzana Makpol

**Affiliations:** Department of Biochemistry, Faculty of Medicine, Universiti Kebangsaan Malaysia, Jalan Raja Muda Abdul Aziz, 50300 Kuala Lumpur, Malaysia

## Abstract

Sarcopenia is a geriatric syndrome that is characterized by gradual loss of muscle mass and strength with increasing age. Although the underlying mechanism is still unknown, the contribution of increased oxidative stress in advanced age has been recognized as one of the risk factors of sarcopenia. Thus, eliminating reactive oxygen species (ROS) can be a strategy to combat sarcopenia. In this review, we discuss the potential role of vitamin E in the prevention and treatment of sarcopenia. Vitamin E is a lipid soluble vitamin, with potent antioxidant properties and current evidence suggesting a role in the modulation of signaling pathways. Previous studies have shown its possible beneficial effects on aging and age-related diseases. Although there are evidences suggesting an association between vitamin E and muscle health, they are still inconclusive compared to other more extensively studied chronic diseases such as neurodegenerative diseases and cardiovascular diseases. Therefore, we reviewed the role of vitamin E and its potential protective mechanisms on muscle health based on previous and current *in vitro* and *in vivo* studies.

## 1. Sarcopenia: A Progressive Geriatric Syndrome

The term “sarcopenia” has been used to describe the progressive loss of muscle structure and function which is associated with increasing age. The origin of “sarcopenia” is from Greek's words, “sarx” and “penia,” which means “poverty of flesh” [1]. Later, geriatricians found that the definition of sarcopenia is inadequate and suggested that the definition of sarcopenia should include the classification of target groups, guidelines to identify high risk patients, and a purpose definition of sarcopenia [2]. Therefore, a consensus definition of sarcopenia was recommended by International Working Group on Sarcopenia. They define sarcopenia as age-associated loss of skeletal muscle mass and function, which could be due to disused muscle, endocrine shift, chronic diseases, inflammation, or nutrient deficiency. Thus older patients with symptoms of declining physical fitness, strength, or overall health can be diagnosed with sarcopenia. Further analysis on body composition using dual energy X-ray absorptiometer (DXA) is needed if patients are bedridden, unable to rise from a chair independently, or with gait speed less than 1.0 m/s. The diagnosis of sarcopenia is confirmed in an individual with gait speed less than 1 m/s and low muscle mass; for example, appendicular fat lean mass relative to height^2^ is less than 7.23 kg/m^2^ in men and less than 5.67 kg/m^2^ in women. Cachexia is included as part of sarcopenia even though they are two different conditions [2]. The European Working Group on Sarcopenia in Older People (EWGSOP) stated that an older person (age of more than 65 years old) with low muscle mass and either low muscle strength or low physical performance (gait speed ≤ 0.8 m/s) can be diagnosed with sarcopenia [3].

Skeletal muscle mass and strength will gradually decrease each year. For example, about 1 to 2% of skeletal muscle mass declines each year after the age of 30 years [4]. It has been reported that the percentage of muscle mass degeneration is higher in men than in women, at a rate of decline of around 12.9% and 5.3%, respectively, for each decade [5]. Besides the decline in muscle mass, loss of muscle strength is also obvious. Longitudinal studies showed that older adults lose their leg strength around 10 to 15% each decade before their age reaches 70 years, and a more dramatic loss was observed in advanced age, in a range of 2 to 4% per year [5, 6]. Furthermore the declined muscle strength is more severe than the progressive shrink of muscle mass [6, 7].

Both men and women can suffer from sarcopenia but the prevalence is higher in men [8]. The prevalence of sarcopenia reaches 5 to 13% in older people aged between 60 to 70 years old [9] and it increases in individuals with age of more than 80 years. The progressive loss of muscle strength in women, however, is slower than in men as reported in various studies [5, 6]. A certain degree of divergence was observed between different populations in the world. For instance, about 53% of men and 31% of women in Caucasian population were diagnosed with sarcopenia [8], while a lower prevalence was shown in Taiwan population with values of 26% for men and 19% for women over 80 years old who are sarcopenic [10].

Sarcopenia results in disability, hospitalization, and death [2]. About 14% of elderly aged between 65 and 75 years old lose independence on daily works and nearly 45% of them require support in their daily life when their age reaches 85 years [11]. The severe muscle mass loss and low muscle strength which occur in the elderly aged 85 years old will further lead to frailty resulting in failure in handling even the easiest task [8]. It has been reported that sarcopenia is one of the main contributors for frailty in older people [12, 13]. Frailty is a condition of high susceptibility for unfavorable prognosis including falls, hospitalization, loss of independence, and eventually death.

The duration of hospital admission has been shown to be correlated with hand grip strength of the elderly whereby individual with stronger hand grip strength has shorter hospitalization period [14]. Data from Aging and Longevity Study in the Sirente Geographic Area, Italy (ilSIRENTE Study), showed that older individuals with sarcopenia have three times higher risk of falling during the 2-year follow-up period as compared to nonsarcopenic individuals [15]. Other studies reported a higher risk of mortality among sarcopenic individuals in a 7-year follow-up period [16], while both quadriceps and grip strength have been shown to be correlated with mortality [17]. These findings strongly indicated that sarcopenia increases vulnerability and leads to poor prognosis in aged population.

Current world life expectancy has increased as compared to last century. Latest report forecasts individuals who were born in year 2000 may live until their age reaches 100 years old [18]. Consequently, world aging population will increase as a result of longer lifespan and lower birth rate, especially in developed nations which are expected to have a spectacular increment in the year 2050 (United Nations 2011). Therefore solution to tackle the problems arising from an aging population has to be obtained, especially in relation to debilitating age-related diseases [18]. Researchers predict that the number of sarcopenic individuals will increase fourfold in 40-year time to more than 200 million patients suffering from sarcopenia [3]. The alarming number of sarcopenia patients will not only affect the health and life status of the population but will also increase the healthcare costs of the country. In the year 2000, 1.5% of healthcare expenses in the United States (US) were for the management and treatment of sarcopenia which were around 18.5 billion dollars for both genders [19]. This condition imposed an economic burden but is potentially modifiable if the prevalence of sarcopenia is reduced. With the global aging population drift, sarcopenia has become a major health problem that could not be ignored. Therefore efforts to discover novel interventions which can concurrently prevent and delay the onset and progression of sarcopenia are important.

## 2. Pathogenesis of Sarcopenia

**Figure 1:**
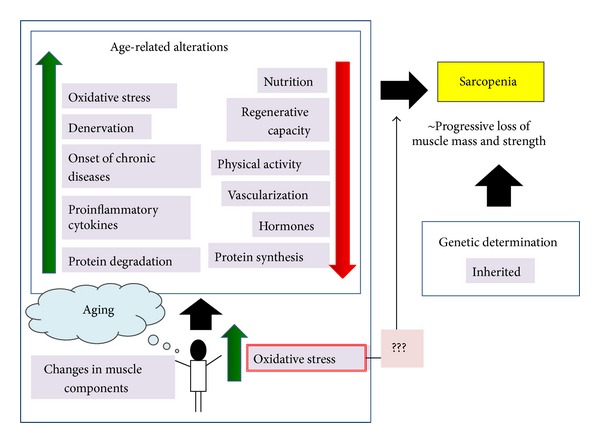
Schematic diagram of the risk factors underlying the progression of sarcopenia. Despite genetic determination, most of the age-related changes are modifiable and can be the target in preventing sarcopenia. Among these factors, increased oxidative stress in aging is more likely to modulate a bunch of signaling cascades that will lead to sarcopenia.

The underlying cause of sarcopenia remains unknown. Although some researchers claimed that environmental factors play the biggest role during advanced age, the influence of genetics has to be considered [20].  Figure 1 illustrates the various possible underlying factors that contribute to the onset of sarcopenia as reported in several studies.

The search for “sarcopenia genes” has drawn the attention of many researchers. Researches have been extensively done to identify candidate genes for sarcopenia in different populations [21]. Amongst the genes that have been identified to play important roles in the pathogenesis of sarcopenia are hormone and receptors genes such as vitamin D receptor (*VDR*) and androgen receptor (*AR*); growth factors and cytokines genes such as ciliary neurotrophic factor (*CNTF*), myostatin (*MSTN*), and insulin-like growth factor 1 (*IGF1*); structural and metabolic genes such as angiotensin I converting enzyme 1 (*ACE*) and alpha actinin 3 (*ACTN3*) [21]. However, these genes are still inconclusive as candidate gene/s that will accelerate the onset of sarcopenia.

Reallocation of fat mass from fat-free mass (FFM) concurrently occurs with decreased muscle power and fitness in advanced age [22, 23]. The muscle fibers in older adults become thinner and shorter, which cause loss of muscle strength [24]. Type II muscle fibers loss has been reported to significantly contribute to the decline in muscle strength during old age [25].

Skeletal muscle aging is very much associated with loss of muscle regenerative capacity [26]. The number of satellite cells that are responsible for muscle repair declines in the elderly aged above 70 years old [27]. However, aged progenitor cells which were exposed to young systemic environment regain their regenerative capacity [28] indicating loss of permissible environment is imperative to cause age-related shift on muscle rejuvenation rather than the number of satellite cells [29]. There is also the possible role of myogenic regulatory factors (MRFs) which affect the severity of sarcopenia. Increased MRFs expression was observed in senescent muscle which is associated with increasing severity of sarcopenia [30]. These changes could be a compensation mechanism of muscle in response to the loss of muscle mass. In contrast other findings reported a decreased MRFs expression at the early stage of senescent cells differentiation and smaller myotubes were formed from senescent myoblasts suggesting early conditions occurring during muscle aging and its potential to develop into sarcopenia [31].

Changes in the nervous system have been considered as one of the causes of failure in muscle power generation during aging [32]. Different expression of neuromuscular junction genes and proteins has been observed in sarcopenic rats, which explains the link between denervation and sarcopenia [33].

Changes of muscle constituent are another important cause of sarcopenia which is interrelated with other factors such as food intake, lifestyle, and chronic diseases including diabetes mellitus and cardiovascular diseases [34]. Other factors may include hormonal changes and the presence of proinflammatory cytokines [35, 36]. High level of proinflammatory cytokines such as interleukin 6 (IL-6) and tumor necrosis factor (TNF) has been reported to reduce muscle mass and strength [36].

Redox and protein imbalance present in advanced age have been reported to cause age-related muscle wasting. Its mechanism is complicated and involves interactions at gene and protein levels. Increased oxidative stress in aging can damage the DNA, protein, and lipid in the muscle. An association between serum protein carbonyls and handgrip strength shown in recent study indicated that oxidative damage affects muscle strength [37]. Sarcopenia may also result from deteriorating anabolic responses in which protein degradation is higher than protein synthesis. At rest, the synthesis rate of myofibril mass is at 0.05% for every hour and this rate increases with nourishment or physical activity in young adults. Similar results, however, were not observed in older adults [38, 39].

## 3. Role of Oxidative Stress on Human Skeletal Muscle Aging

Skeletal muscle can be a privileged site for oxidative damage as it is the organ with the highest consumption of oxygen in the body. Thus, the correlation between the status of cellular antioxidant defense system and muscle injury can be substantial. Under normal conditions, the resistance of skeletal muscle cells towards oxidative stress is higher than other cell types, such as fibroblasts. Young fibroblast cells that were treated with 150 *μ*M H_2_O_2_ once or 75 *μ*M H_2_O_2_ twice in a fortnight developed into stress-induced premature senescence (SIPS) [40]. However, a higher dosage of H_2_O_2_ (1 mM) was required for premature senescence induction in myoblasts [41]. This may indicate that skeletal muscle has a more established antioxidant defense system which is able to quench a high level of reactive oxygen species (ROS).

Increased ROS or other free radicals in skeletal muscle during exercise were first reported in 1982 [42]. However, ROS production is also increased in the muscle after prolonged immobilization and causes disused muscle atrophy [43]. The contradictory findings observed in these two conditions perhaps could be explained by the different mechanisms involved. For instance, different intensity and duration of ROS production influence the final outcome. A reasonable production of ROS in skeletal muscle in a short period is able to activate cellular defense machinery which provides further protection against oxidative stress. However, different mechanisms are engaged when there is a substantial increase of ROS over a long period of time, resulting in increased protein catabolism and decreased cell survival instead of activation of cellular defense mechanism [44]. Increased free radicals level in aging is congruent with the prooxidative effect of exercise, resulting in a prominent elevation of oxidative stress as expected in aged muscle. Studies have proven that the biomarkers of oxidative stress increased in both resting and exercise stages in the muscle of older adults [45]. Similarly, in a rodent model, free radicals that are released from isolated mitochondria are higher in older group, either with or without contraction induction [46]. However, reports on the mechanism of antioxidant defense system in the muscle of aged individuals are still inconsistent and not well explained. A few findings indicate decreased enzymatic antioxidant system in the muscle of old individuals as shown by decreased catalase and glutathione transferase activities in the elderly [47, 48], suggesting lower oxidative capacity in aged skeletal muscle. In contrast, increased superoxide dismutase (MnSOD) and catalase activities were observed in old individuals as compared to young [49].

Recent reports have shown increased oxidative damage on protein, lipid, and DNA in human skeletal muscle with increasing age which is congruent with increased lipid peroxidation, protein carbonylation, and DNA damage [45, 47, 49]. The major free radicals that are present in muscle fibers are superoxide anion and nitric oxide which affect the mitochondria, sarcoplasmic reticulum, sarcolemma, cytosol, and transverse tubules [50]. Mitochondria become the main target of oxidative attack, as they act as the primary ROS production factory in the cells. In 1972, the mitochondria theory of aging postulated that increased oxidative stress in aging is responsible for cellular damage [51]. Continuous oxidative attack to the mitochondrial DNA (mtDNA) and other proteins can cause damage and results in mitochondrial dysfunction [52]. Therefore, aging is very much related to age-associated decline in mitochondrial function. Several studies have shown that the degree of mtDNA damage in skeletal muscle is associated with increasing age [49, 53] and changes in mtDNA are linked to the abnormality in aged muscle [53, 54]. Recently, Szczesny and colleagues [55] reported that declined muscle regenerative capacity during sarcopenia is the result of myoblasts vulnerability to oxidative damage. Oxidative insults will disturb the process of mtDNA repair and decrease the viability of myoblasts. Accumulation of incomplete mtDNA repair products in the mitochondria leads to cell death. Since this defect occurs mainly in growing myoblasts, skeletal muscle regeneration capacity is affected and results in sarcopenia [55].

The imbalance between ROS production and antioxidant defense responses modulates the expression of many transcription factors, responsible for shifting protein synthesis to protein degradation and leading to muscle wasting [50, 56]. Accumulation of ROS will activate mitogen-activated protein kinase (MAPK): extracellular signal-regulated kinase (ERK) and p38 [57]. These MAPKs act as the initial contact between cellular surface and nucleus. ROS will induce the signaling cascade of this pathway resulting in a change in skeletal muscle mass and function. MAPK signaling pathway during resting and exercise suggests a degree of divergence between young and old individuals [58]. Older adults have shown higher resting MAPKs, phosphorylating ERK 1/2, p90RSK, Mnk 1, p38, and JNK/SAPK as the muscle is under stress insults, even during the resting state. However, resistance exercise increased phosphorylation in young subjects, but not in old subjects [58]. Principally, ERK is responsible for cell survival, because it can regulate c-Myc, activator protein 1 (AP-1), and B cell lymphoma-2 (Bcl-2) [59], while p53, nuclear factor-*κ*B (NF-*κ*B), and activating transcription factor 2 (ATF2) are the targets for p38. Both NF-*κ*B and AP-1 are important for the activation of cellular adaptive mechanisms. However, these two transcription factors were not activated in the muscle of old mice [60], suggesting the presence of compromised adaptive responses.

NF-*κ*B is involved in both exercise and disuse-induced skeletal muscle adaptive mechanism. In normal condition, NF-*κ*B presents in the cytosol and forms a complex with I*κ*B protein. In the presence of oxidative stress, I*κ*B-*α* kinase (IKK) will activate and phosphorylate the I*κ*B proteins, unleashing NF-*κ*B, followed by dimerization (with p50 and p65) and nuclear translocation [61]. NF-*κ*B will then activate several genes involved in cell growth, apoptosis, stress responses, and inflammatory processes [62]. NF-*κ*B is also involved in muscle differentiation as indicated by the formation of p65/p50 heterodimer complex which binds to the repressor YinYang1 (YY1) and halts the myogenesis process [63]. Increased NF-*κ*B, p50, and Bcl-3 expression was observed in the muscle of unloaded rats indicating the activation of NF-*κ*B by p50 in disused muscle atrophy [64]. Another study using a transgenic mouse model also reported a similar activation of NF-*κ*B resulting in induction of muscle atrophy [65]. In a muscle-specific transgenic expression of activated I*κ*B kinase *β* (MIKK) mice model, whereby the IKK*β* protein is constitutively active (SS177/181EE mutant), a severe muscle wasting phenotype was observed in both muscles of the limb and trunk, as well as a decline in muscle mass with increasing age [65]. The role of NF-*κ*B in the muscle is further proven by using knockout mouse IKK2^mko^ which is the first NF-*κ*B muscle-specific knockout mice model that has been developed [66]. These mice showed enhanced physical performance and increased resistance to the denervation-induced atrophy [66].

There are contradictory findings reported regarding the effects of exercise where it can either promote or suppress the activation of NF-*κ*B signaling pathway. In a study using rodent, increased NF-*κ*B was reported in the exercised group but decreased in the unexercised group [67]. A concurrent reversible decline of IKK and I*κ*B-*α* in the cytoplasm was observed, whereby the NF-*κ*B signaling cascade was attenuated with antioxidant treatment [67, 68]. Another study showed that high level of NF-*κ*B during aging can be suppressed by regular exercise [69], while acute fatiguing exercise is able to decrease NF-*κ*B activity in human muscles [70]. These findings indicated that NF-*κ*B plays an important role in the muscle as it can be activated or suppressed which results in either muscle wasting or muscle regeneration.

A significant correlation between ROS, mtDNA damage, and TNF-*α* [71] was reported. TNF-*α* stimulates ROS production and may directly cause mitochondrial dysfunction through its receptors [72]. TNF-*α* is also responsible for NF-*κ*B and MAPKs activation [73]. At the same time, increased free radicals may act as second messenger which induces TNF-*α* production. It can be postulated that a cycle exists between TNF, mitochondria, and ROS [74]. In myotubes, activation of NF-*κ*B enhanced IL-6 production [75] and resulted in inflammation which is extensive during aging. Since elevated levels of TNF-*α* and IL-6 are associated with total muscle quality and quantity [36], the role of proinflammatory cytokines on muscle adaptive mechanism in association with redox imbalance may be crucial in the development of sarcopenia.

**Figure 2:**
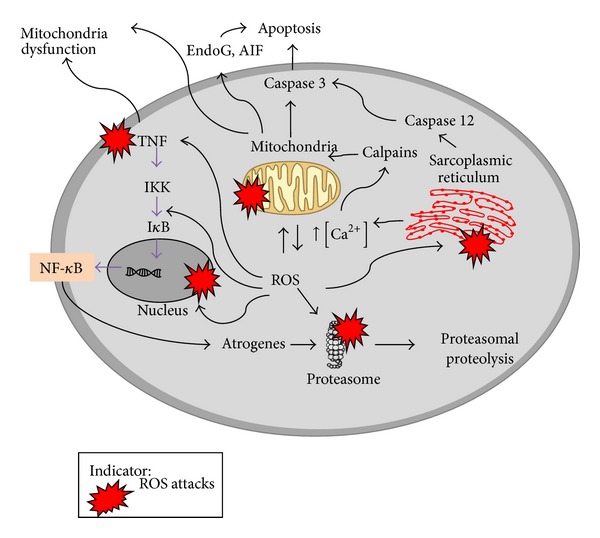
Effects of reactive oxygen species (ROS) on muscle cells. Accumulation of ROS will affect organelles and cell membranes. Alteration on genes and proteins expression leads to muscle wasting during aging. The important mechanisms involved are apoptosis and proteolysis.

How does increased activation of skeletal muscle NF-*κ*B in aging lead to muscle atrophy? NF-*κ*B induces muscle breakdown by promoting proteolysis in skeletal muscle. A previous study using a transgenic mice model has shown that NF-*κ*B increased murine ring finger-1 (MuRF-1) by binding to its promoter, supporting the notion of NF-*κ*B regulated ubiquitin-proteasome pathway (UPS) [65]. This pathway can be regulated by ROS and contributes to muscle mass loss [76]. Furthermore, p38 which is affected by ROS was found to increase the expression of E3 ubiquitin ligases (atrogin-1) in myotube [77]. In senescent muscle cells, the expression of E3 ubiquitin ligases, atrogin-1, and MuRF-1 can either increase [78], remain unchanged [79], or decrease [80]. Even though inconsistent results were reported, their roles in sarcopenia are still worth exploring. Researchers suggested that Akt and FOXO may contribute to the decrease of atrogenes expression in the muscle old rats [80]. However, atrogin-1 and MuRF-1 at transcription level may not represent their actual protein concentrations [81]. Findings on the degradation of oxidized protein without ubiquitination in proteasome 20S served as evident that these atrogenes play an important role in accelerating proteolysis in response to oxidative stress [82]. In addition, reactive nitrogen species (RNS) can activate NF-*κ*B and its cascade response on UPS and matrix metalloproteinases (MMPs), as well as degrading muscle-specific proteins in L6 myotubes [83]. Protein catabolism that occurs during oxidative stress via the activation of NF-*κ*B and other proteins leading to muscle wasting is summarized in Figure 2.

Another cellular process prominent in aged skeletal muscle that leads to muscle atrophy and causes elderly to be more susceptible to sarcopenia is apoptosis [84]. Apoptosis occurs following the activation of p53 by ROS, which further induces Bax to promote apoptosis via the activation of procaspase 3 to caspase 3 [85]. Bax translocates into the mitochondria membrane, releasing cytochrome c and activating caspase 3 [86, 87]. Caspase 3 has also been reported to play an important role in disused muscle atrophy [88]. However, a different apoptosis pathway was suggested in a study involving young and old rats as no significant results were obtained with cytochrome c, Bax, and Bcl-2 expression [89]. Increased apoptosis-inducing factor (AIF) and increased caspase 12 in aged muscle were observed in another study indicating that apoptosis during aging is promoted via the activation of mitochondria independent pathway [90]. Another important protein that may contribute to sarcopenia is endonuclease G (EndoG). EndoG is a mitochondria-specific nuclease which is elevated in old skeletal muscle but not in young skeletal muscle [91]. The apoptotic pathway that will be activated in the muscle depends on the type of muscle fiber. It has been demonstrated that high level of TNF-*α* in aging generates an antiapoptotic response in soleus muscle, but proapoptotic response is generated in vastus lateralis muscle [92]. Thus, further studies are required to elucidate the apoptotic events in aged muscle leading to sarcopenia. Findings from a study on apoptotic events in satellite cells derived from aged adults showed that, compared to young satellite cells, aged satellite cells are more vulnerable to apoptosis at all time points of the experiment (4 to 72 hours) with upregulation of* CASP6* and* CASP9* genes, while* FADD *gene is downregulated leading to decreased survival rate of satellite cells which results in decreased muscle regenerative capability [93] and finally muscle wasting.

Activation of MAPK can cause cell death as MAPK regulates calpains, a cysteine protease, and causes cell death through calcium dependent pathway, leading to sarcopenia [94, 95]. An overall increase in calpain activities which is associated with muscle aging was observed in a study involving 3- and 24-month-old rats [94]. This observation suggested the role of calcium (Ca^2+^) dependent proteolytic pathway in the development of sarcopenia. Apart from the Ca^2+^ channel, ryanodine receptor 1 (RyR1) in sarcoplasmic reticulum may also be involved in the proteolytic pathway since its opening capacity is affected by oxidative stress. This can be explained by an elevated concentration of Ca^2+^ with increasing age in both skeletal muscle and satellite cells [48]. Also the loss of muscle function in the aged group correlated with the permeability of RyR1. This RyR1 channel is more susceptible to oxidation during aging as compared to young rodents [96]. Whilst there is substantial evidence to support a role of ROS in the progression of sarcopenia, the search continues on ways to reverse or prevent this undesirable physiological change during aging.

## 4. Management of Sarcopenia

Elucidating the underlying mechanisms of sarcopenia may aid in discovering potential interventions which will attenuate the decline in muscle mass and strength in advanced age. The potential interventions of sarcopenia are summarized in Table 1.

Since immobilization or inactivity leads to muscle wasting, exercise or increased physical activity may be an effective approach to improve muscle performance. Exercise intervention such as progressive resistance exercise training (PRT) and low intensity exercise is able to provide positive outcomes on the functions of skeletal muscle of the elderly [97, 98]. By engaging exercise, senior subjects have been shown to improve their balance and decrease their falling rate [99, 100]. Nevertheless, running also exerts neuroprotective effect on exercised muscle as well as modulating the expression of growth-related and muscle breakdown-related genes [98, 101].

A systematic review on the role of testosterone and growth hormone replacement therapies has shown the possibility of replacement therapy in improving muscle endurance in older people [102]. However, concern arises in relation to the safety of these therapies. One of the reported adverse effects is the significant correlation between testosterone replacement therapy and formation of prostate cancer as reported by Baltimore Longitudinal Study on Aging which reported a positive correlation between free testosterone level in the blood with aggressive prostate cancer among older men [103].

Other pharmacological interventions that have been used to combat sarcopenia include treatment with caloric restriction mimetic and angiotensin-converting enzyme inhibitors. Introducing rapamycin as an antisarcopenic agent is also beneficial because low level of mammalian target of rapamycin (mTOR) protein has been reported to cause muscle atrophy in aged Fischer 344 X Brown Norway (F344BN) rats [104]. Also, the usage of angiotensin-converting enzyme inhibitors in preventing age-related physical fitness declining has been documented [105].

Nutrient intervention is another potential strategy to prevent sarcopenia [106]. Older adults normally have lower food intake and are more vulnerable to inadequate nutrient consumption [107]. Therefore, modifying the dietary practice of the elderly based on their body requirement may help in improving their muscle performance. For example, malfunction of anabolic system in the aged group reduced the need for increased protein consumption [108]. Furthermore, the role of vitamin D in the conservation of muscle mass and strength can be imperative. Genetic polymorphisms in the vitamin D receptor (VDR) have been linked to sarcopenia occurrence [109] and supplementation of vitamin D decreases the rate of falls among older adults [110].

While diet and exercise alone can be beneficial in improving physical functions, combining nutrient supplementation and exercise is recommended in determining their synergistic effects on sarcopenia [97]. Combination treatment may involve increased protein intake coupled with exercise. A significant increase in muscle protein synthesis in the group with high consumption of protein and performing resistance exercise was reported compared to the group with high protein intake alone [111]. This finding is supported by findings from another study which showed an increment in leg muscle mass in a group with physical exercise and nutrient intervention [112].

Another factor that can be considered in preventing sarcopenia is the central cause of aging which is redox imbalance in the muscle cells. Elimination of ROS may provide a rational solution for sarcopenia prevention particularly in aging as it is a condition where the antioxidant capacity decreases. Therefore, researchers have introduced antioxidants as potential intervention for sarcopenia [113]. However, at present, direct evidence that showed the relationship between antioxidant supplementation and muscle aging prevention is scarce, even though a study has shown that vitamins E and C supplementation in aged rodents was able to improve oxidative stress status and muscle function [114]. Another study reported that an antioxidant cocktail which consists of rutin, vitamin E, vitamin A, zinc, and selenium was able to restore the defective leucine stimulation of protein synthesis and function of aged rats, which triggers the application of antioxidants for sarcopenia prevention [115].

## 5. Vitamin E: A Potential Intervention for Sarcopenia

Vitamin E is a lipid soluble vitamin that exerts antioxidant properties, by scavenging ROS and boosting cellular antioxidative capacity to reduce oxidative damage. It consists of two subgroups which are known as tocopherols and tocotrienols. Both groups have similar fundamental chromanol head but different phytil tail with a saturated phytil tail for tocopherols and unsaturated for tocotrienols. There are four isomers of tocopherol and tocotrienol which are *α*, *β*, *γ*, and *δ* [116] depending on the number and position of methyl groups. Vitamin E is a potent peroxyl radical scavenger that prevents propagation of free radicals in cell membranes and in plasma lipoproteins. The less reactive tocopheroxyl radical produced will react with vitamin C, thereby recycling the vitamin E to its reduced state [117]. Many researches have shown that vitamin E does not only act as an antioxidant but also act as a signaling molecule. However, its action on signaling pathways is still not well explained [118] and studies are ongoing [119, 120]. The interactions between vitamin E and genes in aging and inflammatory age-related diseases have been documented in many studies, whereby supplementation of vitamin E protected against oxidative stress and inflammation [120]. Even though the role of vitamin E in maintaining muscle health per se is rarely mentioned in the literature, a study has shown the role of vitamin E in repairing dystrophic muscle [121].

Vitamin E can be beneficial for aging prevention and treating infections, atherosclerosis, cardiovascular diseases, cancer, diabetes mellitus, and neurodegenerative diseases [120]. The positive effects of vitamin E in aging have been shown in a randomized, double-blinded, placebo-controlled study. A daily dose of 160 mg Tri E tocotrienol when given to 64 participants aged between 37 and 78 years old for 6 months was found to reduce free radical damage as shown by a decreased percentage of DNA damage, sister chromatid exchange frequency (SCE), and urinary 8-hydroxy-2′-deoxyguanosine (8-OHdG) levels in aged group (>50 years old) [122]. In the same study, supplementation of Tri E was found to improve health markers especially by decreasing protein damage and improving HDL cholesterol level besides reducing serum advanced glycosylation end products (AGEs) in adults aged above 50 years old [123]. Interestingly, another study reported a correlation between AGEs level and muscle strength among Japanese men. A significant association between increased AGEs and lower muscle strength was observed which requires further clarification [124]. As vitamin E has been found to reduce AGEs, supplementation could lead to delaying or preventing muscle wasting in the elderly.

The association between vitamin E level and sarcopenia has been reported in various studies, as indicated by grip and knee strength. A prospective population-based study of older adults in Italy done between 1998 and 2000 was known as Invecchiare in Chianti (aging in the Chianti area; InCHIANTI) study. In this study, a total of 1156 subjects aged between 65 and 102 years old were recruited using multistage stratified method, whereby 986 participants without daily vitamin supplementation and capable of conducting physical performance and knee extension strength tests were included for analysis. In an unadjusted analysis, vitamin E daily intake level has been shown to positively correlate with knee extension strength and total physical performance score [125]. After the data was adjusted according to plasma concentration and daily intake, a strong correlation was found between *γ*-tocopherol and muscle strength, as well as a significant correlation between *α*-tocopherol and both measurements [125]. However, final analysis on the correlation between vitamin E and frailty was carried out on 827 participants in InCHIANTI study after excluding the disabled subjects, subjects with cancer, and subjects with vitamin E supplementation [126]. Since sarcopenia is one of the main contributors to frailty in aged populations, hence the correlation between vitamin E and frailty may indicate the role of vitamin E in sarcopenia. Ble and colleagues found a significantly low circulating vitamin E level in frail individuals as compared to nonfrail individuals, suggesting a higher load of free radicals attack in frail individuals [126].

To date, research findings have shown that a satisfactory supply of dietary vitamin E is essential for maintaining muscle health. Although vitamin E deficiency is seldom reported in human, but, in animal models, severe *α*-tocopherol deficiency has been found to affect muscle performance [127, 128] which indicates the importance of vitamin E in protecting muscle from oxidative damage during aging. Muscle morphological changes due to vitamin E deficiency have been observed under electron microscope, including mitochondrial degeneration, sarcoplasmic reticulum fragmentation, and accumulation of myelin figures indicating the progression of muscle dystrophy [121]. As a result, various studies have been carried out to further elucidate these histological observations. Aiming to elucidate the effect of aging and antioxidant deficiency on mitochondrial respiratory chain function in skeletal muscle, a chronic deprivation of vitamin E in rodent model was developed [127]. In this study, rats were fed with a vitamin E-deficient diet for 12, 24, 36, and 48 weeks before their gastrocnemius muscle was obtained for analysis. Data from this study showed accelerated aging in vitamin E-deficient rats as compared to normal rats. In normal rats, increased lipid peroxidation and membrane fluidity of the mitochondria have been observed together with a decrease in cytochrome oxidase and NADH coenzyme Q_1_ reductase indicating greater ROS and lipid peroxides production, as well as impaired mitochondria respiratory chain function during aging. Skeletal muscle, however, starts to show structural abnormality after 12 weeks of exposure to vitamin E-deficient diet. The lipid peroxidation level increases, while declined GSH/GSSG ratio was observed, indicating that the vitamin E-deficient rats are experiencing severe oxidative stress insults. The decline in mitochondria respiratory chain function is more obvious in vitamin E-deficient rats suggesting that vitamin E deficiency may accelerate the aging process [127].

With current technologies, researchers are able to identify the changes caused by vitamin E deficiency, especially at the molecular level to facilitate better understanding of the mechanisms involved. In 2006, Nier and colleagues [128] used Fisher 344 rats to determine the effects of vitamin E deficiency at the transcriptome level. Total RNA was isolated from the musculus quadriceps of the hind leg at 5 time points (days 17, 91, 191, 269, and 430 of deficient vitamin E diet). Results showed that more than 56 genes were differentially expressed in response to vitamin E deficiency at 4 consecutive time points (day 91 to day 430). Most of the genes that were differentially expressed encode for proteins responsible for muscle structure, extracellular matrix, growth and development, oxidative stress, inflammation, and protein degradation. These genes were upregulated during vitamin E deprivation, whereby the severity was time dependent. Nier and colleagues [128] suggested that a defense mechanism has been evoked to protect the muscle structure from oxidative stress during vitamin E deficiency. In another model using *α*-tocopherol transfer protein knockout mice, increased sarcolipin and ubiquitin carboxyl-terminal hydrolase 1 mRNA was observed in ataxia muscle, indicating the effects of vitamin E deprivation on muscle contraction and protein degradation [129]. These findings provide further insight into the molecular mechanisms of vitamin E in the maintenance of skeletal muscle strength and function.

More evidences are emerging to confirm that deficiency of vitamin E leads to muscle atrophy. In other words, supplementation of vitamin E may aid in reversing damaged muscle or at least in maintaining muscle function.

## 6. Protective Mechanisms of Vitamin E against Muscle Aging

Several* in vivo* and* in vitro* studies using different models of muscle aging have provided data on the protective action of vitamin E against muscle aging. Recently, a study showed that vitamin E (tocotrienol-rich fraction (TRF)) can reverse senescence in a stress-induced presenescence (SIPS) model of myoblasts [130] indicating the potential therapeutic effects of vitamin E on muscle cells, although further studies are required to confirm the mechanism involved. In another study, Howard and colleagues [131] have shown that vitamin E aided the restoration of myoblasts membrane, rather than protecting it from laser-induced membrane disruption. However, this poses questions on actual mechanisms involved.

**Figure 3:**
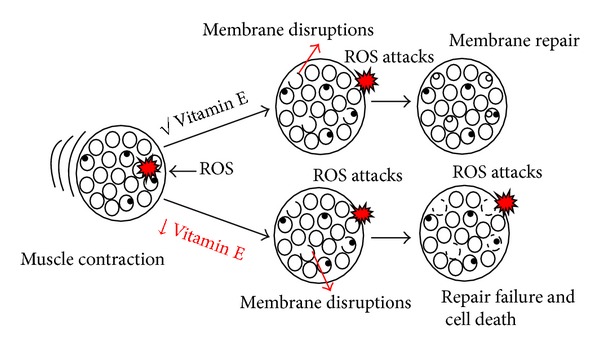
Role of vitamin E on cell membrane repair. During muscle contraction, production of ROS may cause membrane injury which is repaired by vitamin E [131].

In the presence of adequate vitamin E levels, skeletal muscle survived, even though there is a massive production of ROS during muscle contraction because vitamin E helps in repairing the myoblasts membrane (Figure 3). In a study using C2C12 myoblasts, Howard and colleagues indicated that the antioxidant property alone is not sufficient to repair the injured myoblast membrane. *α*-Tocopherol has been reported to be better than other antioxidants as it acts as a stabilizer for the membrane due to its nature lipid soluble properties that allow it to enter the hydrophobic core of plasma membrane. In addition, its chromanol-head group with antioxidant activity can bind to phospholipids head on the membrane surface and scavenges the ROS effectively [131]. As reported earlier, membrane fluidity increases with increasing age, which causes instability of membranes [127]. Therefore, vitamin E acting as a “stabilizer” of the plasma membranes will equally be effective in repairing the damaged membrane during aging as well as in sarcopenic muscle. Besides preventing lipid peroxidation, vitamin E is able to induce Ca^2+^-triggered fusion events that are involved in membrane repair by stimulating the negative impulsive bend [131].

In human studies, vitamin E has been shown to affect muscle strength of the elderly [126]. Several studies have shown the positive effects of vitamin E in reversing muscle damage during extensive muscle contraction (exercise) in healthy men. Vitamin E supplementation at a dose of 800 IU for 28 days resulted in lowering the expression of oxidative stress markers after a downhill run in both young and older men [132]. In another study, a longer supplementation period (12 weeks of vitamin E supplementation) lowered creatinine kinase level after exercise in young men, whereas older men showed decreased lipid peroxidation in both resting state and after exercise, indicating that vitamin E promotes adaptation against exercise induced-oxidative stress and reduced muscle damage [133]. In animal models, similar results were obtained. Increased swimming performance and endurance capacity were achieved after vitamin E supplementation [134, 135] besides attenuation of the early onset of muscle weakness in the elderly [134].

Besides being a powerful antioxidant, vitamin E has been shown to act as an anti-inflammatory agent. The role of ROS and inflammatory processes in inducing muscle atrophy has been suggested in several studies. Aoi and colleagues [136] used* in vitro* L6 myotubes and exercise-induced rats for their* in vivo* model demonstrated that myotubes which were exposed to oxidative stress (H_2_O_2_) showed increased nuclear translocation of NF-*κ*B as well as increased expression of chemokines (cytokine-induced neutrophil chemoattractant-1 (*CINC-1*) and monocyte chemoattractant protein-1 (*MCP-1*)). However, these elevations were prevented by vitamin E treatment. In rat models without vitamin E supplementation, increased neutrophils infiltrated tissues were observed as indicated by elevated neutrophil enzyme myeloperoxidase (MPO), while elevated nuclear translocation component, p65, was demonstrated in gastrocnemius muscle cells. In the vitamin E-supplemented rats, however, significant improvement was seen in MPO, thiobarbituric acid-reactive substances (TBARS), and p65 protein expression after exercise [136] indicating that vitamin E supplementation decreases the activation of cytokines and adhesion molecules. In addition, the expression of a key protein, NF-*κ*B, was attenuated and preventing muscle damage [136]. A more recent report showed that vitamin E reduces inflammation in muscle wasting through NF-*κ*B activation in lipopolysaccharide- (LPS-) induced mice [137] where male* Balb/C* mice were divided into four groups receiving placebo-saline, placebo-LPS, vitamin E-saline, and vitamin E-LPS. Their findings showed that LPS significantly upregulated IL-6 gene and increased protein expression in skeletal muscle. However, vitamin E lowered the expression of IL-6 at both gene and protein levels suggesting that vitamin E may regulate pretranslation level and modulated IL-6 expression as its expression was halted at the mRNA stage. Similar findings were observed for IL-1*β*, whereby vitamin E attenuated the LPS induced increment of IL-1*β* at both gene and protein levels. The positive effect of vitamin E supplementation was also observed in the regulation of TNF-*α* and NF-*κ*B expression [137]. As previously reported, NF-*κ*B was able to stimulate the expression of IL-6 in myotubes [75]. Vitamin E may halt the overall proinflammatory cytokines responses by suppressing NF-*κ*B and thus offering benefits to the population that is suffering from inflammation, such as the elderly [137]. As inflammatory responses affect muscle mass and strength [36], these findings may indicate that muscle weakness in the elderly can be ameliorated by vitamin E through NF-*κ*B suppression.

Mitochondrial dysfunction and apoptosis are very much related to ROS and muscle wasting which are affected by vitamin E supplementation in protecting against skeletal muscle atrophy. Studies have been carried out using male Charles River mice to investigate the effectiveness of vitamin E in maintaining mitochondrial membrane integrity and preventing mitochondrial dysfunction and apoptosis induced by severe high-altitude hypobaric hypoxia in skeletal muscle [138, 139]. These findings demonstrated that vitamin E attenuated the impact of hypoxia on mitochondrial function and apoptotic signaling pathways. Vitamin E prevents mitochondrial alteration, as well as decreasing Bax expression and improving Bcl-2/Bax ratio in skeletal muscle [138, 139]. The apoptotic pathways involving calpains and caspases are also modulated by vitamin E which counteracted muscle wasting. Vitamin E was reported to prevent increases in *μ*-calpains expression and caspases activity (caspase 3, caspase 9, and caspase 12) in unloading-induced rats which further reduced apoptosis in a stressful condition and thus prevented muscle atrophy [140].

Apart from apoptosis, proteolysis is likely to be involved in the onset of sarcopenia.* In vitro* study using proteolysis-inducing factor (PIF) and angiotensin II- (Ang II-) induced myotubes has shown rapid increment of ROS production and protein degradation in muscle cells. These unfavorable elevations, however, were attenuated by *α*-tocopherol [141]. Vitamin E inhibited phosphorylation of I*κ*B and suppressed NF-*κ*B activation, which finally attenuated protein degradation. These findings supported the role of ROS, NF-*κ*B, and UPS in the progression of muscle atrophy [76, 141, 142], where NF-*κ*B may be the central transcription factor that is regulated by vitamin E in protecting skeletal muscle against sarcopenia.

Another study demonstrated the ability of vitamin E in counteracting unloading-induced soleus muscle atrophy [140] indicated by decreased severity of muscle atrophy with vitamin E treatment. Vitamin E prevented the decline in type I and type II muscle fiber mass and decreased the level of TBARS, promoted heat shock protein 72 (HSP72) expression, and downregulated proteolysis-related genes. Induction of HSP72 protein by vitamin E induced protective mechanism against muscle wasting and promoted repair of oxidative damaged proteins [140]. These findings also supported the data reported by Russell and colleagues [141], which showed vitamin E prevented proteolysis by decreasing MuRF-1 expression which was elevated during unloaded *γ*-tocopherols induction [140].

In a study involving a diabetic model, myoblast cells were exposed to high glucose medium for 14 days to induce diabetes [131]. Vitamin E reversed the impaired membrane repair system after diabetes induction as indicated by decreased membrane disruption. These findings are in parallel with the findings from another study which showed that *γ*-tocopherols prevented glucose oxidase-induced insulin resistance in L6 myotubes [143]. As mentioned previously, diabetes mellitus may be one of the underlying causes of sarcopenia [34]. Thus the promotion of membrane repair and increased insulin sensitivity by vitamin E are important in diabetes mellitus which further prevent the development of myopathy.

The role of another antioxidant vitamin C in muscle repair has been reported in several studies. Vitamin C exerts its effect by regenerating vitamin E and subsequently maintains the level of vitamin E, which is responsible for muscle protection [131]. A cocktail of antioxidants which consists of vitamin C, vitamin E, and *α*-lipoic acid improved exercise perfusion after 5 minutes of plantar flexion exercise as well as improving muscle oxidative capacity in the elderly. These findings showed that oxidative stress contributes to age-related decline in skeletal muscle perfusion during physical activity which is improved by antioxidants [144]. In animal studies, vitamins E and C have been shown to exert a protective effect in aged rodents. An improvement in oxidative indices of chronically loaded muscle of aged F344BN rats was observed with vitamins E and C supplementation [114] indicating that combination of vitamins E and C may provide a stable antioxidant supply in aged muscle, subsequently reducing muscle atrophy.

In contrast, other researchers reported that vitamin E alone or in combination with vitamin C did not alter the unfavorable responses in the muscle during exercise [145–147]. In young healthy men, supplementation of vitamin E at a dose of 1200 IU for 30 days did not normalize the elevated creatinine kinase, torque deficit, and the extent of Z-band disruption caused by contraction-induced muscle damage [145]. Eccentric exercise resulted in muscle damage and poor muscle performance and affected blood redox balance and haemolysis despite vitamins E and C supplementation raising a query on the needs of antioxidant supplementation in healthy individuals for protection against muscle damage [147].

In short, several studies have suggested the possible pathways of vitamin E protection against muscle atrophy. Vitamin E is able to combat oxidative stress and stabilized the plasma membrane in protecting the muscle against oxidative insults. Interestingly, the nonoxidative way of vitamin E in preventing inflammation, proteolysis, and apoptosis is also described in various skeletal muscle wasting models.

However, there are contradictory results as well reporting the effects of vitamin E supplementation against muscle atrophy with no or little improvement seen on physical performance indicating inconsistency of findings due to discrepancy in subject recruitment and vitamin E supplementation. A supplementation study showed that 1000 IU of vitamin E given to young and old men who did downhill running did not affect the exercise-induced response in a consistent manner [146]. This finding highlighted the variation in the metabolic system of different age groups. Furthermore, the definition of vitamin E which only includes *α*-tocopherol in most studies provides unexplored research area especially in skeletal muscle aging for the other 7 isomers of vitamin E especially tocotrienols.

## 7. Future Prospects of Vitamin E in the Prevention and Treatment of Skeletal Muscle Aging

Most of the studies that have been carried out used *α*-tocopherols as a representative of vitamin E. However, it did not reflect other possible effects of different isomers of vitamin E in protecting against muscle atrophy and muscle aging. The beneficial effects of tocotrienols beyond tocopherols have been reported in several studies [116]. Hence, the role of tocotrienols in muscle aging prevention should not be ignored.

Comparison between tocopherols and tocotrienols has been carried out on their antioxidant and nonantioxidant properties. Tocotrienols have been reported to be a more potent antioxidant compared to tocopherols due to their structural differences which facilitate cell membrane penetration [148, 149]. Tocotrienols may have higher recycling efficiency and cellular uptake than tocopherols [148, 150]. These features may contribute to tocotrienol superior effectiveness in certain conditions. Corresponding to this, Mazlan and colleagues [151] reported that a lower dose of *γ*-tocotrienol was able to protect cells from H_2_O_2_-induced apoptosis, while *α*-tocopherol requires higher dose to preserve cell viability. Among the evidences which highlighted the importance of tocotrienols in muscle aging prevention is a study that compared the effects of *α*-tocopherols and tocotrienol-rich fraction (TRF) in forced swimming rats. TRF-treated rats have shown better physical performance and oxidative status than *α*-tocopherol-treated rats, as indicated by longer swimming period and better antioxidants profile in the muscle [135]. Hence, a certain degree of divergence may be present between the different isomers of vitamin E in their actions on muscle aging. This will further initiate exploration on the molecular mechanism of tocotrienols or TRF in preventing sarcopenia and muscle aging.

The molecular and cellular events modulated by vitamin E in protecting muscle from aging are still unknown. Even though there are studies which reported the possible genes and pathways modulated by vitamin E in various cell types [119], data are still limited, especially studies on skeletal muscle cells. Therefore, more pieces of research should be carried out to explore the role of vitamin E at molecular level in finding novel genes or elucidating the role of NF-*κ*B [141] and HSP72 [140] that has been shown to be affected by vitamin E in the muscle. These proteins may influence the inflammatory and adaptive pathways, which contribute to aging as well as age-related muscle atrophy. With the development of new technologies, molecular research can be very exciting. Recently the role of epigenetics has drawn the focus of many researchers in exploring the molecular mechanism of muscle aging. In skeletal muscle, epigenetics may control or regulate myogenesis. For instance, interaction between Myb-binding protein 1a (Mybbp1a) and miR-546 has been reported to suppress myoblast differentiation [152]. Thus, it is possible that protection of muscle function by vitamin E could be via epigenetics modulation.

## 8. Conclusion

Sarcopenia is a geriatric syndrome, which is characterised by progressive loss of muscle mass and strength. It may lead to unfavorable consequences, including morbidity and mortality. Understanding the risk factors and its underlying mechanisms is imperative to manage this disease, even though the theory of oxidative stress in aging is well established.* In vivo* and* in vitro* studies have demonstrated that increased free radicals in aging are a major contributor to sarcopenia development. Thus, the rationale of introducing antioxidants, such as vitamin E, in the prevention and treatment for sarcopenia is justified, even though further studies are required to elucidate its molecular effects.

Vitamin E, either as single isomer or in combination, can be introduced as an antiaging intervention for the muscle. The reduced level of vitamin E in the body has been reported to be associated with increased risk of muscle atrophy. Current evidences indicated that vitamin E can prevent muscle atrophy and promote muscle regeneration, although further investigations are required to confirm molecular mechanisms involved. Other effects of vitamin E, including modulation of signaling pathways, have been proven indirectly in other cell types initiating studies to elucidate molecular effects and antisarcopenic properties.

Most of the studies reported using *α*-tocopherol as a representative of vitamin E which does not reflect the actual effects of various forms of vitamin E in protecting against muscle atrophy and muscle aging. Hence, the role of tocotrienols in muscle aging prevention warrants further investigation.

## Figures and Tables

**Table 1:** The potential interventions for sarcopenia.

	Treatments	References
Exercise	PRT aerobic (running)	[97] [98]

Hormone replacement	Testosterone Growth hormone	[102]

Pharmacological intervention	CR mimetics, for example, rapamycin Angiotensin-converting enzyme inhibitors, for example, enalapril	[104] [105]

Diet and nutrition	Protein Vitamin D Antioxidants, for example, vitamins E and C	[108] [110] [114, 115]

Combination	Diet and exercise	[111, 112]

*CR: caloric restriction; PRT: progressive resistance exercise training.
